# Corticothalamic Pathways From Layer 5: Emerging Roles in Computation and Pathology

**DOI:** 10.3389/fncir.2021.730211

**Published:** 2021-09-09

**Authors:** Rebecca A. Mease, Antonio J. Gonzalez

**Affiliations:** Institute of Physiology and Pathophysiology, Medical Biophysics, Heidelberg University, Heidelberg, Germany

**Keywords:** pyramidal neurons, corticothalamic, higher-order thalamus, bursting, thalamus, layer 5, neural coding, pathology

## Abstract

Large portions of the thalamus receive strong driving input from cortical layer 5 (L5) neurons but the role of this important pathway in cortical and thalamic computations is not well understood. L5-recipient “higher-order” thalamic regions participate in cortico-thalamo-cortical (CTC) circuits that are increasingly recognized to be (1) anatomically and functionally distinct from better-studied “first-order” CTC networks, and (2) integral to cortical activity related to learning and perception. Additionally, studies are beginning to elucidate the clinical relevance of these networks, as dysfunction across these pathways have been implicated in several pathological states. In this review, we highlight recent advances in understanding L5 CTC networks across sensory modalities and brain regions, particularly studies leveraging cell-type-specific tools that allow precise experimental access to L5 CTC circuits. We aim to provide a focused and accessible summary of the anatomical, physiological, and computational properties of L5-originating CTC networks, and outline their underappreciated contribution in pathology. We particularly seek to connect single-neuron and synaptic properties to network (dys)function and emerging theories of cortical computation, and highlight information processing in L5 CTC networks as a promising focus for computational studies.

## Introduction

The thalamus is a bilateral structure of the diencephalon that serves integral roles in a significant range of neurophysiological functions including sensory information relay, learning and memory, motor control, and regulation of sleep and wakefulness (Herrero et al., 2002; Yuan et al., 2016). Positioned above the midbrain, the thalamus displays widespread connectivity with the cerebral cortex, as well as subcortical and temporal structures (e.g., mammillary bodies, fornix, and hippocampus), acting as a central hub for functional brain networks (Hwang et al., 2017). The thalamus is traditionally fractionated into functional nuclei, each of which participates in feedback and/or feedforward communication with unique cortical areas (Morel et al., 1997; Yuan et al., 2016; Halassa and Kastner, 2017).

**FIGURE 1:**
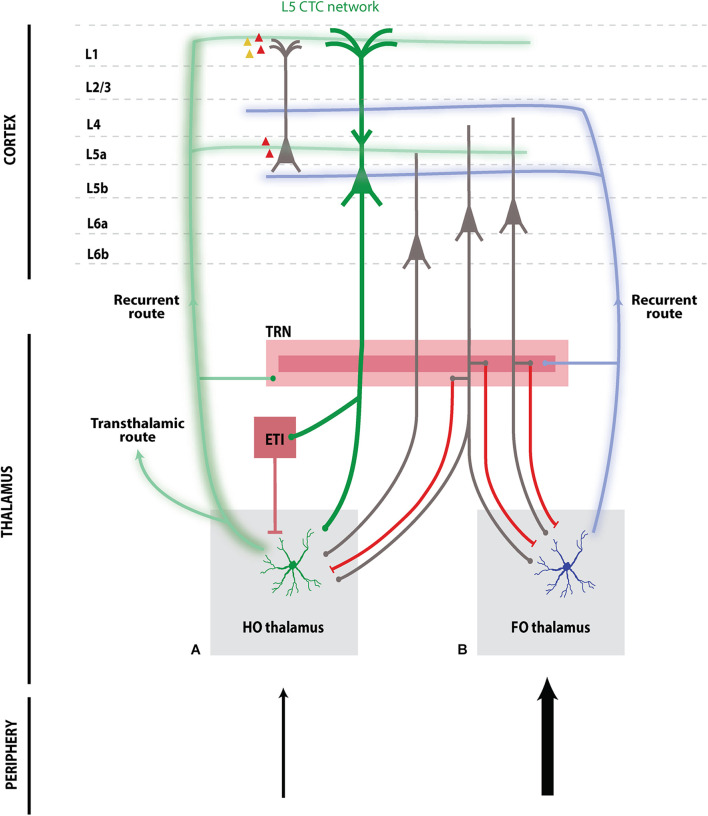
Schematic of higher-order **(A)** and first-order **(B)** cortico-thalamo-cortical networks. L5 thick-tufted pyramidal cells in L5B send projections to HO thalamic nuclei and to additional subcortical targets including extrathalamic inhibitory (ETI) sources, e.g., the ZI and APT, which exert strong inhibition on HO nuclei. HO thalamus **(A)** sends TC projections across transthalamic routes to other cortical regions, or forms a recurrent route back to the same cortical region targeting L5A and upper layers. HO TC projections target excitatory pyramidal cells, as well as PV (red) and 5HT3-positive inhibitory (yellow) interneurons, although there are modality-specific variations (e.g., associative thalamus). In contrast, FO thalamus **(B)** sends recurrent TC projections to cortical layers L5B and L4. L6 corticothalamic cell subpopulations display distinct connectivity with thalamic nuclei: Upper L6A cells specifically target FO thalamus, while lower L6A cells target both FO and HO thalamus; both populations send collaterals to inhibitory TRN. L6B cells specifically target HO thalamus but do not send collaterals to TRN. Both HO and FO nuclei engage intrathalamic inhibitory feedback loops via excitatory projections to TRN. Outer “shell” TRN sends inhibitory projections to HO thalamus while inner “core” TRN sends inhibitory projections to FO thalamus.

The thalamus is massively innervated by deep corticofugal cells in layer 5 (L5) and layer 6 (L6) (Figure 1). While these dense projections have been documented since the early 20th century (Cajal, 1906), in-depth study of the pathways and their functional impact on the thalamus has only recently become experimentally accessible (Guy and Staiger, 2017). Indeed, technological advances in high-yield electrophysiology as well as cell-type-specific optogenetics and anatomical tracing have elucidated interactions between specific cortical layers and thalamic nuclei (Luo et al., 2018). In particular, such approaches have refined our understanding of “higher-order” (HO) thalamic nuclei – those regions of the thalamus that are strongly innervated by cortical L5 pyramidal neurons (Sherman, 2007; Figure 1A). However, this understanding still lags that of “first-order” (FO) thalamic nuclei (Figure 1B), which do not receive L5 input and have proven more tractable with typical sensory physiological experiments.

A recent study provides beginning evidence that L5-HO thalamus connectivity may represent a “default” scheme: while each sensory modality (e.g., somatosensation, vision, and audition) retains a distinct FO and HO nucleus, FO and HO nuclei are each genetically homologous across modalities. Intriguingly, the transcriptional identity of FO nuclei depends on the presence of peripheral input, as neonatal ablation of these inputs leads to FO nuclei transitioning to a HO transcriptional program and descending cortical input that is typically HO-directed (Frangeul et al., 2016). In addition, recent studies have highlighted the importance of L5-originating CTC networks in diverse functions, such as permitting selective thalamic-driven modulation, dynamic cortical coupling, sending motor instructions, and informing higher cortical areas about which motor instructions have been issued, as well as roles in learning and plasticity in cortical networks (Sherman and Guillery, 2011; Gambino et al., 2014; Audette et al., 2019; Usrey and Sherman, 2019). However, a synthesized view of HO CTC computation is still elusive, given the diverse nonlinear single-neuron and synaptic properties of these networks, and complex connectivity with other subcortical regions.

In this focused review, we discuss foundational and recent studies of L5-originating HO CTC networks across cortical regions. In particular, we emphasize findings distinguishing HO CTC networks from better-studied FO regions of thalamus that serve as bottom-up relays to the cortex. Many excellent existing reviews summarize the recent rapid progress in understanding anatomical and functional properties of CTC networks (e.g., Usrey and Sherman, 2019; Shepherd and Yamawaki, 2021), particularly contrasting FO and HO CTC connectivity. For context, we review this material but focus mainly on studies relevant to understanding computation within HO CTC networks as well as the emerging importance of these networks in learning and pathology.

**FIGURE 2:**
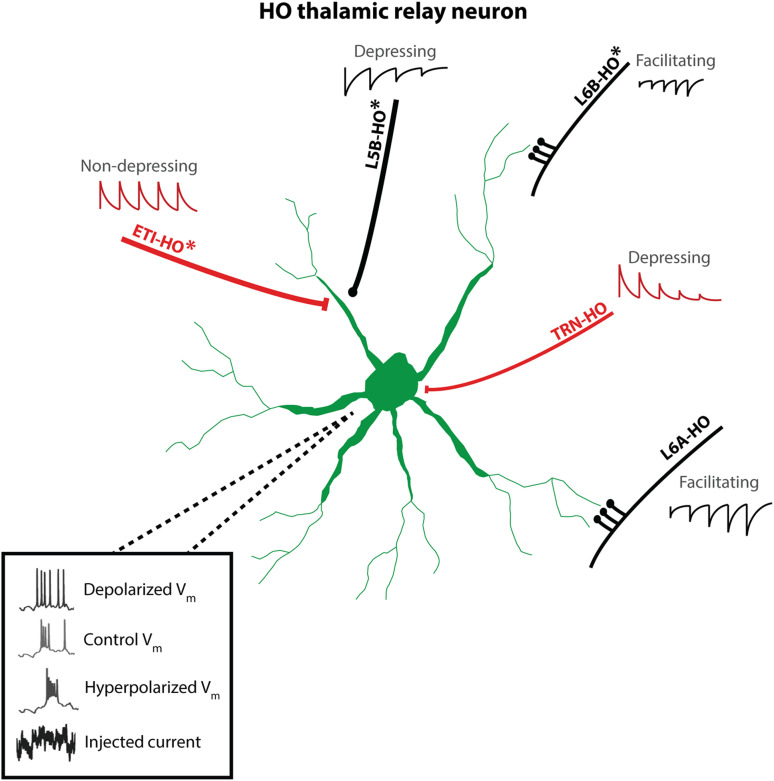
Cartoon of synaptic inputs to HO thalamic relay neurons and intrinsic excitability patterns. Asterisks (*) indicate inputs which target HO thalamus and are not present in FO nuclei. L5tt neurons provide strong, depressing “driving” excitatory input to proximal dendrites via large synapses, while L6A CT *en passant* synapses provide relatively weaker, facilitating, modulatory excitatory input to distal dendrites. L6B provides *en passant* excitatory inputs (synaptic dynamics not yet reported). Inhibitory TRN inputs show strong depression. In contrast, powerful inhibitory ETI synapses are located in close proximity to L5tt excitatory inputs and show little depression. Not shown: additional area-specific sources of driver input reported in “convergence” zones of HO thalamus and feedforward inhibitory loops L5tt-ETI-thalamus and L6A-TRN-thalamus. Inset voltage traces: HO thalamic neurons show characteristic voltage-dependent bursting: depolarization inactivates bursting mechanisms and promotes tonic spiking.

Our aim is to present a unified yet comprehensible overview of these increasingly appreciated circuits that is accessible to experimental and computational neuroscientists from outside the thalamocortical field. The schematic in Figure 1 outlines the networks of interest and Figure 2 illustrates available information on synaptic inputs to HO thalamus. Key literature is organized according to modality and methodology (Table 1) and relevance to pathology (Table 2). This review is structured as follows:

1. L5-HO thalamus definitions and network connectivity schemes.
2. HO thalamic nuclei across modalities.
3. Inhibitory control of HO thalamus.
4. Synaptic properties of L5-HO thalamus projections.
5. HO thalamus intrinsic properties and single-cell information processing.
6. HO encoding of L5 cortical information.
7. HO thalamocortical innervation of cortex and roles in sensory processing and cognition.
8. Clinical relevance of L5-originating CTC networks.

**TABLE 1:** Key literature describing anatomical and/or physiological properties of CTC networks across sensory modalities and an example non-sensory modality.

	Somatosensation	Vision	Audition	Cognitive/non-sensory
Origin of L5tt CT projections	S1	V1	A1	PFC
HO thalamic nucleus	POm	LP/pulvinar	MGBd	MD
Reported cortical targets	S1, S2, M1	All visual cortical areas	All auditory cortical areas	PFC
CT anatomy (L5 to HO thalamus)	Hoogland et al., 1991; Bourassa et al., 1995; Veinante et al., 2000; Groh et al., 2008, 2013; Theyel et al., 2010; Guo et al., 2017, 2020; Sumser et al., 2017; Mo and Sherman, 2019; Prasad et al., 2020; Sampathkumar et al., 2021	Bourassa and Deschenes, 1995; Li et al., 2003c; Masterson et al., 2009; Stitt et al., 2018; Bennett et al., 2019; Prasad et al., 2020; Blot et al., 2021	Ojima, 1994; Bartlett et al., 2000; Rouiller and Welker, 2000; Llano and Sherman, 2008, 2009; Williamson and Polley, 2019; Pardi et al., 2020	Xiao et al., 2009; Collins et al., 2018; Prasad et al., 2020; Anastasiades et al., 2021
L5-HO synaptic/intrinsic physiology	Reichova and Sherman, 2004; Landisman and Connors, 2007; Groh et al., 2008; Theyel et al., 2010; Seol and Kuner, 2015; Mease et al., 2017; Guo et al., 2020; Desai and Varela, 2021	Li et al., 2003a, b; de Souza et al., 2019; Desai and Varela, 2021	Desai and Varela, 2021	Collins et al., 2018; Anastasiades et al., 2021
TC anatomy	Koralek et al., 1988; Lu and Lin, 1993; Bureau et al., 2006; Groh et al., 2010; Meyer et al., 2010a; Theyel et al., 2010; Wimmer et al., 2010; Viaene et al., 2011; Audette et al., 2017; Casas-Torremocha et al., 2019; Sermet et al., 2019; El-Boustani et al., 2020; Rodriguez-Moreno et al., 2020	Saalmann et al., 2012; Stitt et al., 2018; Bennett et al., 2019	Pardi et al., 2020	Delevich et al., 2015; Collins et al., 2018; Mukherjee et al., 2020; Anastasiades et al., 2021
TC physiology	Bureau et al., 2006; Lee and Sherman, 2008; Petreanu et al., 2009; Theyel et al., 2010; Audette et al., 2017; Casas-Torremocha et al., 2019; Mo and Sherman, 2019; Sermet et al., 2019; El-Boustani et al., 2020; Guo et al., 2020	Purushothaman et al., 2012; Stitt et al., 2018	Lee and Sherman, 2008; Pardi et al., 2020	Delevich et al., 2015; Collins et al., 2018; Anastasiades et al., 2021
L5 CTC function *in vivo*	Groh et al., 2013; Gambino et al., 2014; Mease et al., 2016a,b,c; Rojas-Piloni et al., 2017; Audette et al., 2019; Williams and Holtmaat, 2019; Zhang and Bruno, 2019; LaTerra et al., 2020; Suzuki and Larkum, 2020; Takahashi et al., 2020; Pagès et al., 2021	Bender, 1983; Purushothaman et al., 2012; Saalmann et al., 2012; Stitt et al., 2018; Yu et al., 2018; Bennett et al., 2019; de Souza et al., 2019; Blot et al., 2021; Kirchgessner et al., 2021	Asokan et al., 2018; Williamson and Polley, 2019; Pardi et al., 2020	Parnaudeau et al., 2013; Schmitt et al., 2017; Rikhye et al., 2018; Mukherjee et al., 2020

*Recent studies using cell-type-specific approaches are emphasized.*

**TABLE 2:** Summary of key literature findings on the relevance of higher-order CTC pathway components across pathological states including pain, tinnitus, and neuropsychiatric disorders.

Relevant pathology	Authors	Findings
Chronic pain	Cichon et al. (2017)	• Chronic pain (SNI model) elicits hyperactivity in L5 cells in S1; correlates with degree of mechanical allodynia • Reduction in PV and SOM interneurons; increase in VIP interneurons • DREADD-based activation of SOM interneurons prevented development of mechanical allodynia; activation of PV interneurons not effective
	Masri et al. (2009)	• Animal model of central pain syndrome • Spinal cord lesions elicited higher spontaneous firing rates and responsiveness to innocuous/noxious stimulus in the PO • Lower spontaneous firing rate in ZI, causing PO disinhibition
	Whitt et al. (2013)	• Spinal cord lesions elicited higher spontaneous firing rate and magnitude/duration of responses to noxious stimuli in the MD
	Meda et al. (2019)	• Optogenetic activation of MD-ACC pathway in SNI and chemotherapy-induced neuropathy mice models produced a conditioned place-aversion • Same effect observed following direct inhibition of L5 ACC-MD projection
	Saadé et al. (2007)	• Inactivation/lesions of MD nucleus reduced thermal and mechanical hyperalgesia in neuropathic pain model
Tinnitus/noise-induced damage	Asokan et al. (2018)	• Noise-induced damage to cochlear afferents elicits hyperactivity of L5 projection cells in the auditory cortex for several weeks • May drive hyperexcitability and strengthened coupling in tinnitus-associated brain networks
Neuropsychiatric disorders	Kim et al. (2011)	• Hypoxic-like damage in PFC enhanced MD/PFC theta-frequency coherence and burst frequency of MD neurons • T-type calcium channel knockdown decreased theta-frequency coherence and attenuated associated symptoms (e.g., frontal lobe seizures

Throughout, we attempt to highlight missing experimental data and theoretical perspectives.

## L5 to Higher-Order Thalamus: Corticothalamic Connectivity From the Pyramidal Tract

Pyramidal neurons in L5 of the cortex serve as the major exit point by which cortical signals are directed to subcortical circuits. Aside from L6 corticothalamic (CT) neurons, only L5 neurons are reported to make synapses outside of the neocortex; in contrast to the L6A CT pathway which is restricted largely to thalamus, L5 neurons project to several subcortical targets, including thalamus, superior colliculus, pons, brainstem, and spinal cord. Despite its complexity and computational power, these L5- and L6-originating outputs represent the only means by which the cortex can influence subcortical processes and thereby influence behavior (Sherman and Guillery, 2013; Usrey and Sherman, 2019; Prasad et al., 2020; Sherman and Usrey, 2021). Indeed, innervation by L5 driving inputs is the criterion for the influential and useful “higher-order” terminology introduced by Sherman and colleagues. Presently, several L5-originating CTC networks have been identified across sensory modalities including somatosensation, audition, and vision (Sherman, 2016; Table 1).

Recently, the development of novel transgenic mouse lines has granted refined experimental access to cell-type- and layer-specific observations (Gong et al., 2007; Gerfen et al., 2013; Harris et al., 2014, 2018; Daigle et al., 2018). In conjunction, advancements in optogenetics and electrophysiology have enabled targeted monitoring and manipulation of neuronal circuits, and dissection of physiological and functional circuit properties. These powerful approaches have begun to disentangle the properties of L5-HO thalamus pathways from L6-thalamus pathways, for example, through the use of *Rbp4, Npr3*, and *thy-1* mouse lines (Groh et al., 2013; Daigle et al., 2018; Guo et al., 2020; Prasad et al., 2020; Kirchgessner et al., 2021), establishing key characteristics of L5 projections to the thalamus. Most importantly, L5 inputs to HO nuclei are sparse and display characteristic “driver” properties, in contrast to L6 projections, which target both HO and FO thalamic nuclei and provide modulatory input (Sherman and Guillery, 2002; Reichova and Sherman, 2004; Sherman, 2016).

Higher-order thalamic nuclei are specifically targeted by L5 “thick-tufted” (L5tt) pyramidal tract neurons in L5B (Harris and Shepherd, 2015; Figure 1A, green). As recipients of information from all cortical layers, L5tt neurons are well-suited for integrating cortical signals–particularly characteristic are the elaborately branched apical dendrites that cover nearly a column in width. Notably, L5tt neurons show minimal axonal branching within the cortex, suggesting a main role is to distribute information subcortically and coordinate behavior (Ramaswamy and Markram, 2015). In line with this predicted function, many anatomical studies demonstrate that L5tt neurons send branching collaterals to subcortical targets including the HO thalamus, brainstem, and spinal cord–even at the level of single L5 neurons innervating more than one subcortical region (Veinante et al., 2000; Sherman, 2016; Guo et al., 2017; Rockland, 2019; Prasad et al., 2020). However, there is also evidence that L5tt subpopulations with distinct intrinsic properties innervate specific subcortical targets (Hattox and Nelson, 2007; Rojas-Piloni et al., 2017). This point raises the intriguing possibility that L5tt populations may have excitability and synaptic properties matched to their innervation targets. L5tt axonal branching is a matter of ongoing study and appears to be species-specific (Smith et al., 2014; Rockland, 2019). Reports also depend on specific experimental methodology, with axonal reconstructions reporting more branching versus retrograde tracing. High-throughput reconstruction efforts will be key to sharpening our understanding of L5tt innervation of subcortical targets in the near future.

### Transthalamic and Recurrent Pathways

From the perspective of the HO thalamus, L5tt projections provide a strong but sparse drive to individual neurons (discussed in the next section). Where does this information go after being further processed in HO thalamus? (Sherman and Guillery, 2011) suggested a significant revision to our understanding of thalamocortical processing with the “transthalamic” hypothesis, specifically that cortical L5tt neurons in one source region send information to a secondary cortical recipient region via HO thalamus. Recent anatomical and functional data suggest that transthalamic pathways paralleling “direct” cortico-cortical pathways could be a common feature of the thalamocortical system and that these pathways carry distinct rather than redundant information (Sherman, 2016; Bennett et al., 2019; Mo and Sherman, 2019; Blot et al., 2021). However, there is recent evidence that certain HO circuits can also be involved in recurrent “closed-loop” networks, in which the same cortical region providing L5tt input to HO thalamus is itself reciprocally innervated by HO thalamus (Wimmer et al., 2010; Guo et al., 2020). This reciprocal connectivity motif is seen in FO CTC networks, where L6A modulatory CT projections originate from the same cortical region innervated by FO TC projections. However, the transthalamic and recurrent network motifs are not mutually exclusive and may subserve different functions. As we highlight below, based on region-specific descending CT and ascending HO TC connectivity patterns, different combinations of recurrent and transthalamic communication are possible. An important focus for future studies is to understand the distinct signals transmitted by transthalamic and recurrent pathways, and to uncover the computational scheme integrating these information channels with cortico-cortical signals.

Currently, it is understood that HO nuclei, which represent a majority of the thalamus by volume, participate in the generation of activity in distinct cortico-thalamo-cortical (CTC) networks (Guillery, 1995; Sherman and Guillery, 2011; Sherman, 2016). Additionally, these CTC networks serve to integrate a diverse range of cortical and subcortical signals (Groh et al., 2013; Bickford, 2015). While a full assessment of this literature is beyond the scope of this review, we note that these “convergence zones” have been reported across modalities (Groh et al., 2013; Bickford, 2015). For example, projections from the primary visual cortex in the HO lateral posterior (LP) nucleus overlap with terminals originating in the superior colliculus (Li et al., 2003c; Masterson et al., 2009). Elsewhere, Bosch-Bouju et al. (2013) introduce the concept of a “super-integrator,” where motor regions of the thalamus integrate information from the cortex with information from the basal ganglia and cerebellum. Additional convergence zones have been identified by exploiting the differential distribution of type 1 and type 2 vesicular glutamate transporters, which are specific to cortical and subcortical inputs, respectively (Rovó et al., 2012; Bickford, 2015). Recent anatomical evidence points to a role for HO thalamus in integrating information from different cortical regions (Prasad et al., 2020), even at the level of single cells (Sampathkumar et al., 2021).

## Higher-Order Nuclei Across Modalities

In this focused review, we center our discussion on HO nuclei (Table 1) involved in primary sensory modalities (somatosensation, audition, and vision), as well as on HO nuclei that have been implicated in pathology (e.g., pain and cognitive dysfunction). A very recent anatomical survey of L5tt-subcortical projections has identified additional HO thalamic regions (Prasad et al., 2020); the functional properties of these projections remain to be characterized.

In the somatosensory system, the medial subdivision of the posterior nucleus (POm) receives L5tt input from the primary somatosensory cortex (S1), and thereafter recurrently targets S1 (Audette et al., 2017; Guo et al., 2020) and relays information trans-thalamically to the secondary somatosensory cortex (S2) (Theyel et al., 2010) and the primary motor cortex (M1) (Mo and Sherman, 2019). As Table 1 reflects, the somatosensory HO CTC circuit has provided a wealth of recent anatomical and functional data on cell-type-specific interactions and the *in vivo* impact of HO thalamus on cortical function.

In the visual system, the pulvinar nucleus (PuV) [or the homologous LP nucleus in rodents] is the L5tt-receiving HO nucleus, and is connected to all visual cortices (Bender, 1983; Saalmann et al., 2012; Bennett et al., 2019; de Souza et al., 2019). While pulvinar has been largely studied in primates, recent cell-type-specific and optogenetic studies have made increasing use of mouse models (Table 1). Regions within the LP that are reciprocally connected to the cortex are dominated by small terminals, while those that lack reciprocal connectivity are dominated by large terminals (Van Horn and Sherman, 2004). LP projections to higher visual areas have been shown to integrate descending information from L5tt cells in V1 with that from several cortical and subcortical areas, and the information conveyed to higher-order visual areas differs from that conveyed by cortico-cortical projections from V1. For example, while intracortical V1 projections convey information to the anteriolateral higher visual area about visual motion, the transthalamic route to this region (i.e., through the LP nucleus) integrates information about visual motion and the animals’ movement (Purushothaman et al., 2012; Stitt et al., 2018; Blot et al., 2021).

In the auditory system, the dorsal aspect of the medial geniculate nucleus (MGBd) receives L5tt input from the primary auditory cortex (A1), and relays to higher-order auditory regions (e.g., A2) (Lee and Sherman, 2011; Lee, 2015; Sherman, 2017). Cortical projections to the inferior colliculus (IC) have axon collaterals in the MGBd, although the degree and functional relevance of this branching is unclear (Asokan et al., 2018; Williamson and Polley, 2019). Notably, detailed physiological characterizations of the L5-MGBd-cortical pathways are lacking (Table 1).

While these sensory HO nuclei have received significant attention, there also exist “associative” L5tt-receiving HO nuclei that participate in non-sensory modalities. Here we emphasize rodent studies in the mediodorsal (MD) nucleus (Table 1), which contributes to learning, memory, and decision-making processes. MD’s influence on cognitive abilities results from interactions with L5tt-projecting frontal areas such as the prefrontal cortex (PFC) (Mitchell, 2015; Bolkan et al., 2017; Schmitt et al., 2017; Alcaraz et al., 2018; Pergola et al., 2018; Rikhye et al., 2018). Indeed, 20% of PFC projections (mainly from dorsal and medial areas) to MD stem from L5 (Xiao et al., 2009). It has been found that PFC output neurons in fact branch to both MD and the functionally diverse ventromedial (VM) nucleus, which may enable synchronized thalamic spiking across nuclei (Collins et al., 2018). In addition, the MD nucleus is implicated in mediating affective and emotional aspects of pain (Whitt et al., 2013; Mitchell, 2015).

## Inhibition of Higher-Order Thalamus

A small but growing body of work demonstrates that HO thalamus is subject to inhibitory effects that either differ or are entirely distinct from those seen in FO circuits. Here we summarize the main points of distinction, and refer readers to Halassa and Acsady (2016) for a more comprehensive treatment of inhibitory control of thalamus.

### Intrathalamic Inhibition of HO Thalamus

A central regulator of thalamic function is feedback inhibition via thalamic reticular nucleus (TRN), a thin layer of GABAergic neurons partially encapsulating the relay nuclei which project to cortex (Pinault, 2004). As well as TC afferents to cortex, thalamic relay neurons also send thalamoreticular projections to TRN which in turn provide feedback inhibition to relay neurons (Figure 1); the temporal scale of this inhibition is sensitive to spiking patterns (Figure 2), with high-frequency bursts triggering long-lasting IPSCs due to GABA “spillover” to extrasynaptic receptors, while tonic spiking patterns trigger shorter IPSCs (Halassa and Acsady, 2016). A recent pair of milestone studies in the somatosensory thalamus reveal that properties of HO and FO intrathalamic inhibitory circuitry differ significantly: HO nucleus POm excites and is inhibited by a discrete shell population of TRN neurons; furthermore, the synaptic dynamics of POm-TRN connections as well as the intrinsic properties of POm-connected TRN neurons are functionally distinct from those in VP-TRN circuits (Li et al., 2020; Martinez-Garcia et al., 2020). Thus, it may be that the dynamics of intrathalamic inhibition are matched to the distinct signal processing requirements of HO and FO circuits carrying L5tt and sensory information, respectively.

Given its role in gating thalamocortical transmission as well as its positional and physiological properties, the TRN has been implicated in the regulation of attention in the “searchlight hypothesis” (Crick, 1984; Crabtree, 2018). Regions in the TRN show increased activity in response to attentional stimuli, and the specific region in which this response is found is modality-dependent (McAlonan et al., 2000, 2006). Moreover, limbic TRN projections correlate with arousal states, while sensory TRN projections are suppressed by attentional states (Halassa et al., 2014). Work by Halassa et al. (2011) demonstrates TRN-dependent control of thalamocortical firing mode and state regulation, where selective drive of TRN causes a switch from tonic to burst firing and generates state-dependent neocortical spindles (Halassa et al., 2011).

Likewise, there is evidence for an attentional role of HO thalamus. For example, the MD is activated in humans during tasks requiring a rule-dependent shift in attentional allocation (i.e., set-shifting), such as the Wisconsin card-sorting task (Monchi et al., 2001; Halassa and Kastner, 2017). Human and monkey studies also point to a role of the pulvinar in visual attention. Pulvinar lesions in patients result in impairments in filtering distracting information, while pulvinar inactivation in monkey impairs spatial attention (Danziger et al., 2004; Snow et al., 2009; Wilke et al., 2010; Halassa and Kastner, 2017). In addition, Yu et al. (2008) describe the pulvinar’s role in sustained attention, employing the five-choice serial reaction time task to show that half of recorded units in this nucleus were attention-modulated (Yu et al., 2018). However, TRN control of HO thalamus in the context of attention and arousal has yet to be systematically investigated.

### L6 CT Inputs to HO Thalamus

Thalamic reticular nucleus inhibition is also influenced by topographically organized CT projections from L6A which innervate both relay neurons and neurons in TRN (Lam and Sherman, 2010; Figure 1). Thus, these CT inputs simultaneously provide direct excitatory input to relay neurons and exert additional top-down control on inhibition via TRN, providing a cortical signal for task or attention-specific modulation of the thalamus. Overall, the net functional impact of L6A CT signals on thalamic relay neurons is determined by an excitation-inhibition balancing act of rate-dependent synaptic depression and facilitation (Crandall et al., 2015); this dynamic balance is seen *in vivo* in both HO and FO nuclei (Kirchgessner et al., 2020). It should be noted that HO and FO nuclei are targeted by partially disjoint populations of neurons in L6A (Thomson, 2010; Whilden et al., 2021; Figure 1), perhaps in keeping with the distinct reticular inhibitory circuits discussed above. Finally, aside from the L6A CT pathways, recent studies have identified a HO-thalamus specific pathway from layer 6B (Figure 1) which does not send collaterals to TRN and appears to exert strong excitatory influences on relay neurons (Hoerder-Suabedissen et al., 2018; Ansorge et al., 2020), although detailed physiological measures of these inputs are currently lacking. Notably, L6B neurons are orexin-sensitive (Bayer et al., 2004), suggesting that the cortex may provide HO thalamus with descending excitation based on wakefulness.

### Extrathalamic Inhibition

Another key differentiator of HO thalamic circuits is the presence of additional subcortical inhibition from sources of “extrathalamic inhibition” (ETI) (Figures 1, 2), such as the zona incerta (ZI) and the anterior pretectal nucleus (APT) [reviewed in Halassa and Acsady (2016)]. Anatomical and experimental studies have suggested that these ETI sources exert a significantly more powerful inhibitory effect on the thalamus than do intrathalamic inhibition sources (Barthó et al., 2002; Lavallée et al., 2005; Park et al., 2018). In contrast to the TRN, which targets all thalamic nuclei, ETI inputs display greater target selectivity, a property that is evolutionarily highly conserved (Halassa and Acsady, 2016). Across sensory modalities, studies have demonstrated that HO, but not FO nuclei, receive ETI input, and this appears to serve as the inhibitory counterpart of L5tt excitatory inputs. This topographical restriction means that these ETI projections do not interact with ascending sensory relays, nor do they directly impact FO thalamus activity. Overall, ET inhibition of HO nuclei is notably strong, rapid, and precise, rendering it capable of influencing the timing of individual spikes in target cells (Halassa and Acsady, 2016). Further spatial selectivity has been established within subregions of the ZI. For example, Lavallée et al. (2005) found that the posterior (PO) HO nucleus receives inhibitory input from the ventral division of the ZI, which is in fact the same ZI subregion that receives input from the APT (Giber et al., 2008; Murray et al., 2010). Further underscoring the importance of ETI nuclei in sculpting HO thalamus processing of L5tt inputs, ZI and APT are also strongly driven by L5tt collaterals (Figure 1)–thus, HO nuclei are embedded in an additional feedforward inhibitory loop similar in motif to the L6A-TRN loop, but specific to HO nuclei and with different synaptic properties (Barthó et al., 2002, 2007; Halassa and Acsady, 2016).

## Properties of PT Corticothalamic Synapses

L5tt-HO thalamus projections form sparse, large glutamatergic synapses electronically close to the soma of target thalamic relay neurons (Figure 2), typically on thick proximal dendrites near branch points (Hoogland et al., 1991; Bourassa et al., 1995; Deschênes et al., 1998; Rouiller and Welker, 2000). Terminals vary widely in size (2–10+μm), notably these distributions include a heavy tail of “giant” synapses reported in somatosensory, visual, and auditory HO nuclei (Llano and Sherman, 2008; Hoerder-Suabedissen et al., 2018; Prasad et al., 2020). “Giant” boutons are glomerular structures containing multiple synapses (Hoerder-Suabedissen et al., 2018) and development of these structures appears to be use-dependent (Hayashi et al., 2021). A recent anatomical comparison of L5tt-HO thalamus terminals (Prasad et al., 2020) reports some pathway-specific variation in size: V1 to pulvinar/LP, and PFC to MD terminals are somewhat smaller than S1 L5tt-POm benchmark “giant” synapses, but still larger than L6A-thalamus terminals.

These “driver” synapses have high release probability and high postsynaptic density of AMPA and NMDA receptors (Hoogland et al., 1991; Li et al., 2003b; Reichova and Sherman, 2004; Groh et al., 2008). While sparse–for example, single L5tt fibers form an average of only three boutons onto single POm neurons–single fiber activation can nevertheless trigger extremely large (∼3 nA in rat, ∼ 800 pA in mouse) excitatory postsynaptic currents (EPSCs), which drive large, unitary EPSPs (>10 mV) (Li et al., 2003b; Groh et al., 2008; Seol and Kuner, 2015). Aside from sheer synaptic strength, the EPSCs have exceptionally fast (<1 ms/2 ms rise/decay in rat) kinetics due to the presence of the GluA4 AMPA subunit (Seol and Kuner, 2015). While strong, L5tt-HO thalamus synapses are characterized by rapid and pronounced frequency-dependent depression due to presynaptic depletion of releasable vesicles (Reichova and Sherman, 2004; Groh et al., 2008; Seol and Kuner, 2015; Figure 2). At the L5tt-POm synapse, frequencies greater than ∼2 Hz induce depression, with paired-pulse ratios of 0.5 at 20 Hz, with similar values reported for L5tt-LP (Li et al., 2003b) and L5tt-MD and L5tt-VM (Collins et al., 2018), although comparable physiological data for the auditory system is lacking. In sum, despite some modality-specific anatomical variations which require further functional characterization (Prasad et al., 2020), L5tt-HO synapses appear to have relatively conserved properties supporting powerful, temporally precise, adaptive information transmission.

## Higher-Order Thalamus Single Neuron Computation

Strong synaptic drive by L5 prompts the question of how HO neurons encode descending cortical information. In the context of neural coding, corticothalamic information transfer has been widely studied as L6A’s modulatory influence on FO neurons’ encoding of sensory drivers, in combination with aligned feedforward inhibition from TRN (e.g., Contreras et al., 1996; Wolfart et al., 2005; Béhuret et al., 2013). In HO nuclei, the situation is different: L5tt driver synapses provide a mechanism by which L5tt neurons’ precise spike times reach HO thalamus with minimal temporal distortion as large, fast EPSCs; via synaptic depression, EPSC amplitude will reflect L5’s preceding spiking history. To generate thalamic spikes, these cortically generated EPSCs must be integrated with other driver inputs (if present, e.g., Groh et al., 2013; Bickford, 2015; Blot et al., 2021), and finally transformed to spikes by intrinsic membrane properties.

### Bursting

Intrinsic thalamic contributions to a corticothalamic code from L5tt to HO thalamus are expected to be considerable, given the nonlinear bursting properties of thalamic neurons (Llinás and Jahnsen, 1982) and increased appreciation for bursting as a significant mode of information processing (Zeldenrust et al., 2018b). In brief, across modalities, HO neurons display the well-known burst-tonic excitability switch characteristic of thalamic relay neurons (Figure 2). Burst spiking–high-frequency sodium APs superimposed on a transient calcium plateau or “low-threshold spike”–arises from the interplay of slow voltage-dependent currents, namely I_*T*_, carried by the Cav3 subfamily of calcium channels, with some contribution from I_*H*_, the hyperpolarization-activated cation current (Jahnsen and Llinas, 1984; Destexhe et al., 1993; Sherman, 2001). In particular, the phenomenon of bursting in thalamic neurons is well-studied, but a limitation to understanding HO bursting is that most detailed biophysical measurements come from FO neurons, typically from the visual system.

Several reports indicate that bursting properties differ between HO and FO nuclei: (Ramcharan et al., 2005) report more bursting in primate HO MD and HO pulvinar; cortico-recipient LP in rat shows enhanced bursting (Li et al., 2003a) and likewise, in tree shrew pulvinar (Wei et al., 2011), increased bursting was associated with increased expression of Cav3.2 and SK2, an afterhyperpolarization-generating K+ channel which can support repetitive firing. The somatosensory system appears to be an exception, as (Landisman and Connors, 2007) found that HO POm spikes at lower frequencies relative to FO VPM, both within bursts and during tonic spiking, associated with a higher voltage threshold for AP initiation in HO POm. A recent comparison across sensory modalities (Desai and Varela, 2021) provides a mechanistic framework for understanding some of these differences based on electrophysiology and simulations. Rebound burst size (number of APs) in HO nuclei was comparable across sensory modalities. However, differences between FO and HO bursting were modality specific and in agreement with data previously reported for single modalities: in visual and auditory thalamus, FO nuclei were less bursty than HO nuclei, while in somatosensory thalamus, the FO nucleus was more bursty than HO. This spectrum of bursting properties could be reproduced by changing the voltage-dependence and maximal conductance of I_*T*_. Taken together, these studies suggest functionally relevant differences in fast and slow intrinsic excitability mechanisms in HO and FO thalamic neurons.

Indeed, a recent comprehensive report of the thalamus transcriptome (Phillips et al., 2019) found that HO and FO nuclei show distinct transcriptomic profiles based on expression of genes tightly linked to neuronal identity (ion channels, receptors), in line with distinct intrinsic excitability of HO neurons. These points underscore the need for additional basic functional data from HO neurons, particularly voltage-clamp studies of biophysical properties necessary for computational studies, and at minimum, caution in using FO models as stand-ins for HO neurons in simulations.

### Intrinsic Transformation of L5tt-HO EPSCs

We emphasize that careful consideration of bursting mechanisms is key to understanding how HO thalamus performs computations on descending cortical signals, as these mechanisms are responsible for nonlinear transformation of L5tt-evoked EPSCs to EPSPs. In the simple case of synaptic input after a period of inactivity, e.g., the beginning of a cortical Up state–large undepressed EPSCs will be further enhanced by activation of I_*T*_; functionally, this means that a single L5tt spike can evoke bursts of spikes in a post-synaptic HO neuron (supralinear corticothalamic spike transfer) (Groh et al., 2008; Seol and Kuner, 2015). In the more complicated case of higher input rates, synaptic depression will decrease EPSC size and preceding depolarization will simultaneously inactivate I_*T*_, supporting a more linear EPSC→ EPSP transformation. Here, integration of coincident inputs from different L5tt neurons can be required to drive output spikes (Groh et al., 2008). Although L5tt neurons’ drive of HO neurons at the single-neuron level has been most studied in the somatosensory system, the parallel effect of synaptic depression paired with inactivation of bursting is also apparent in data from other modalities (Li et al., 2003b; Collins et al., 2018).

The information processing role of bursts is an active area of study, and our understanding of thalamic burst coding continues to be refined. Recent studies (Elijah et al., 2015; Mease et al., 2017; Zeldenrust et al., 2018a; Park et al., 2019) make it clear that various properties of thalamic bursts (number of spikes, timing of spikes, burst onset, etc.) can convey information about presynaptic inputs, so it may be that the distinct bursting properties of HO thalamus subserve a particular functional role. In HO POm, we found that intrinsic bursting and high spiking threshold of POm neurons provides a mechanism for “multiplexed coding” of low- and high-frequency (∼5 Hz and >100 Hz) information, and that high-frequency encoding channel showed information-preserving adaptation (Mease et al., 2017). Furthermore, the exact bandwidth of the “slow” encoding channel was tuned by depolarization. This finding suggests that POm spike trains could carry information both in burst size and precise spike timing within bursts. However, the implications of this intrinsic code in combination with strong L5tt-HO synaptic depression have yet to be assessed. While it is not clear if these findings can be generalized to other nuclei, the observation that HO bursts seem to show less variation across modalities (Desai and Varela, 2021) suggests that further investigation of a common HO thalamic computational scheme for encoding L5tt inputs may provide new insights beyond those gleaned from studies focusing on computation in FO thalamus.

### Neuromodulation

A final unexplored factor in HO thalamic encoding of L5tt inputs is neuromodulation. As membrane potential is a critical mediator of bursting properties, any modulatory input altering baseline depolarization or hyperpolarization would be expected to exert strong control over HO thalamic encoding. While neuromodulation specific to HO thalamus has not been widely studied, evidence suggests that it may dynamically regulate HO CTC network activity. For example, Stitt et al. (2018) suggest that activity in the HO thalamus may be state-dependent and influenced by ongoing levels of arousal. Elsewhere, it was found that the brainstem cholinergic system preferentially suppresses spontaneous activity in the POm-targeting region of ZI, thereby enhancing whisker responses in the POm (Masri et al., 2006; Trageser et al., 2006). Furthermore, Varela and Sherman found neuromodulation may exert differential effects on FO and HO neurons: while all FO and most HO neurons are depolarized by muscarinic and serotonergic activation, a significant fraction (15–20%) of HO neurons are hyperpolarized (Varela and Sherman, 2007, 2009). Taken in combination with attentional control of TRN and arousal-dependent activation of L6B discussed in the previous section, these studies tentatively point to an unexplored state-dependence of HO thalamus’s processing of L5tt drive. Future studies of this topic are warranted, particularly incorporating insight from recent molecular profiling work (Phillips et al., 2019) which may provide specific candidate mechanisms for state-dependent control.

## Higher-Order Thalamic Encoding of L5tt Cortical Information

A wealth of evidence demonstrates that HO thalamus spike output is strongly affected or even contingent upon L5tt output, in line with the driver characteristics of this pathway. Manipulations of S1 cortex (Diamond et al., 1992) showed that spiking in HO POm but not FO VPM depend on S1 input; more specific optogenetic drive of L5 *in vivo* is sufficient to drive large driver EPSPs and bursts in POm, and VGAT optogenetic inhibition of S1 eliminates POm spiking (Groh et al., 2013; Mease et al., 2016c). Similarly, portions of HO LP (Bennett et al., 2019; Kirchgessner et al., 2021) are driven by L5tt inputs from V1. This situation is supported by findings that receptive fields in HO thalamus *in vivo* are typically broad and less specific for primary sensory drive (Moore et al., 2015; Urbain et al., 2015; Mease et al., 2016b; Williamson and Polley, 2019), suggesting that HO nuclei receive most sensory information after it has been processed by L5. Given the relatively low convergence of L5tt neurons onto HO neurons (Sumser et al., 2017; Rockland, 2019), single HO neurons may integrate the spiking of small cortical ensembles; indeed, in anesthetized mice, there is some evidence that HO thalamus may be driven to spike by just 1–3 active L5tt neurons *in vivo* (Mease et al., 2016c). Thus, the question becomes: what particular patterns of L5tt activation could be encoded by HO thalamus?

A critical piece of information necessary for understanding what L5tt signals HO neurons transmit back to the cortex is exactly what L5tt neurons encode and what the “raw” cortical input arriving in HO looks like via single fibers and convergent inputs from groups of L5tt neurons. L5tt neurons have been the focus of intense experimental and theoretical research interest over decades (Larkum, 2013; Ramaswamy and Markram, 2015; Sakmann, 2017), but despite this arguably focused attention, the exact signals propagated through L5tt pathways are not yet fully understood, despite being one of the most active neurons during behavior (de Kock et al., 2007; O’Connor et al., 2010; Senzai et al., 2019). The emerging picture is that L5tt spiking on both single-neuron and population levels carries complex information, with L5tt neurons typically showing broad sensory tuning (de Kock et al., 2007; Williamson and Polley, 2019) in line with their integration of multilaminar information in basal and apical dendrites, which is presumably inherited by HO thalamic targets. Extensive discussion of L5’s coding across modalities is beyond our scope; here we attempt to orient two key characteristics of L5tt output–bursting and ensemble synchrony–to what is known about further subcortical processing in HO thalamus.

### L5tt Bursting

The active, nonlinear dendritic properties of L5tt neurons [reviewed in Larkum (2013); Ramaswamy and Markram (2015)] provide a single-neuron substrate for the integration of “top-down” and “bottom-up” information streams arriving at different lamina. Excitation of either basal or apical dendrites leads to sparse spiking, but near-coincident excitation of both regions triggers a burst of high-frequency (>100 Hz) APs which depends on a backpropagating AP in the soma triggering a calcium plateau in the apical tuft (Larkum et al., 1999; Larkum, 2013). In this framework, bursts indicate a temporal alignment of internal representation and novel external information. Such high-frequency bursts are characteristic of L5tt neurons in the primary somatosensory (Larkum et al., 1999; de Kock and Sakmann, 2008), visual (Shai et al., 2015), and auditory (Llano and Sherman, 2009; Williamson and Polley, 2019) cortices, and are associated with perception (Takahashi et al., 2016, 2020) and exploratory whisker touch (de Kock et al., 2021). While bursting appears to be an important nonlinear computational property of L5tt neurons, it remains to be determined if these spiking patterns are relevant for postsynaptic targets such as HO thalamus, i.e., are these high-frequency patterns faithfully transferred across the L5tt-HO thalamus synapse?

### L5tt Synchrony and Ensemble Activity

Particular patterns of L5tt synchronization often involve inhibitory feedback interactions with interneurons, reviewed in Naka and Adesnik (2016). In particular, L5tt neurons excite both Martinotti cells (a subset of somatostatin-positive interneurons) which inhibit L5tt apical tufts, and PV neurons, which provide perisomatic inhibition to L5tt neurons. Inhibition from Martinotti neurons seems particularly tuned to L5tt bursting, as Martinotti neurons are preferentially recruited by high-frequency inputs from L5tt due to synaptic facilitation. This “frequency-dependent disynaptic inhibition” is a mechanism linking L5tt bursting to population synchrony, as bursting in a handful of L5tt neurons preferentially inhibits and synchronizes neighboring L5tt neurons (Silberberg and Markram, 2007; Hilscher et al., 2017).

Synchronous activity in particular frequency bands is an important mechanism by which cortical circuits transfer information (Buzsáki and Draguhn, 2004) and recent work has begun to relate understanding of cortical rhythms to specific neuronal cell types. For example, Adesnik and Scanziani (2010), Otsuka and Kawaguchi (2021) report that stimulation of L2/3 pyramidal neurons evokes beta/gamma band activity in L5tt neurons. Corticothalamic synchrony has been explored extensively from the perspective of CTL6 inputs to thalamus (e.g., Contreras et al., 1996; Bal et al., 2000; Béhuret et al., 2013), but how L5 synchrony might propagate to the HO thalamus is only beginning to be studied. For example (Stitt et al., 2018) find that alpha and theta band coherence is prominent in deep-layer PPC and pulvinar interactions; while this study did not specifically isolate layer 5, the involvement of HO nuclei suggest that L5tt input could participate in driving these corticothalamic oscillations. In sum, L5 synchronous population oscillations over a wide range (alpha, beta, theta, and gamma) of frequencies suggest that L5tt-HO thalamus synapses will have synchronized activation at diverse temporal scales that will engage different degrees of synaptic depression.

Recent efforts have begun to explore how pyramidal single-cell properties and population activity in the cortex are linked to implement network-level information coding strategies. The recently proposed Burst Ensemble (Naud and Sprekeler, 2018) theory suggests that ensemble event rate in L5tt (spike or burst count/time) reflects somatic input, burst probability reflects apical input, and burst rate reflects coincident somatic and apical input, while a simple spike rate code cannot disambiguate these combinations of inputs. Lipshutz et al. (2020) explored how simple model networks of pyramidal neurons can implement canonical correlation analysis, finding the features in apical and basal inputs which have maximal correlation; in this framework bursts indicate maximal alignment between the two input sources. Lankarany et al. (2019) demonstrate synchrony-division multiplexing: S1 neurons receiving common input can use the rate of asynchronous spiking to encode the intensity of low-contrast stimulus features while using the timing of synchronous spikes to encode the occurrence of high-contrast features. These studies highlight the importance of simultaneously considering cortical bursts and synchronous spikes as putatively informative signals for postsynaptic targets such as HO thalamus.

In sum, L5tt spike trains appear to carry information in spike count and timing and population synchrony; it is not well understood to what degree these information streams are disentangled and further transformed in HO thalamus. Groh et al. (2008) show that stimulating L5tt-POm synapses with *in vivo* L5tt spiking patterns resulted in single L5tt spikes driving POm spiking or bursting after long periods of silence in contrast to subthreshold EPSPs evoked at higher presynaptic L5tt rates sufficient to induce depression. Similarly, Collins et al. (2018) report that PFC L5tt EPSCs depress and only drive spiking in HO MD at the onset of 10 Hz stimulation. However, Groh et al. (2008) found that coincident activation of separate L5tt inputs served to overcome synaptic depression, and suggested a role for HO thalamus in detecting synchronous firing of L5tt neurons. Within the HO thalamus multiplexing framework (Mease et al., 2017), such coincident L5tt upstream activity could be encoded by the timing and spike count of POm bursts. Such coincidence detection may also work similarly in the case of integration of cortical and subcortical drivers, as in anesthetized animals, POm output reflects the latency between L5 activation and whisker stimulation (Groh et al., 2013).

Abundant evidence highlights L5tt bursts as somewhat privileged spiking patterns, both in terms of selective encoding of inputs and intracortical impact. From the point of view of HO thalamus, bursts would be translated into temporally discrete EPSCs of decreasing size. We hypothesized that the intrinsic properties of POm neurons may allow these EPSCs to influence spike timing within HO bursts, a situation which would preserve much of the temporal information with L5tt bursts (Mease et al., 2017), but this possibility remains to be tested particularly in awake animals during which HO thalamus bursting is less pronounced. An alternative hypothesis is that subsequent spikes in L5tt bursts evoke EPSCs too small to drive spiking in HO thalamus, or require coincident bursts from multiple presynaptic L5tt neurons to drive HO spiking. Combining cell-type-specific approaches with depth-resolved high-yield recordings in cortex (e.g., Senzai et al., 2019) and HO thalamus (e.g., Kirchgessner et al., 2020) will likely provide data to test these hypotheses.

## Higher-Order Thalamocortical Projections to Cortex

How do signals computed by HO thalamus functionally impact the cortex? As we have reviewed above, although the exact features in L5tt spiking encoded by HO neurons and sent back to cortex remain to be determined, several recent studies have begun to clarify the function of HO TC inputs within the cortical microcircuit. These insights have built upon foundational anatomy studies demonstrating that HO TC projections follow the “matrix” pattern of TC innervation in that projections are not somatotopically precise and tend to be wide-ranging across cortical areas [reviewed in Harris and Shepherd (2015)]. In this section, we differentiate HO and FO TC pathways and highlight recent advances in understanding cell-type-specific HO TC innervation. We close with a discussion of recent evidence for the functional importance of HO TC inputs in higher-level CTC computations.

The function of HO CTC circuits cannot be viewed in isolation from other components of the cortical microcircuit–we refer to Shepherd and Yamawaki (2021) for a comprehensive review of CTC wiring, functional connectivity, and integration with feed-forward cortico-cortico circuits. Moreover, we also restrict our scope to differentiating HO and FO TC pathways in reciprocally connected circuits (e.g., S1→ POm→ S1). Readers are referred to other sources for discussion of HO properties for identified transthalamic sensory pathways S1-POm-S2 (Theyel et al., 2010; Viaene et al., 2011); there is evidence that TC inputs may have different laminar targets and synaptic properties in transthalamic circuits (e.g., Casas-Torremocha et al., 2019; Rodriguez-Moreno et al., 2020).

In S1, layer-specific HO TC inputs tend to interdigitate with and complement FO TC inputs (Figure 1), with dense HO projections to L5A and L1 (Koralek et al., 1988; Lu and Lin, 1993; Bureau et al., 2006; Meyer et al., 2010b; Wimmer et al., 2010), These distinct innervation patterns predict that HO thalamus provides synaptic input to particular cortical neuronal targets with dendrites in these lamina; the degree of expected TC innervation is often predicted as a function of depth as the TC projection density multiplied by the dendritic reconstruction’s summed cross-section. Optogenetic-aided circuit-mapping methods, (e.g., Bureau et al., 2006; Audette et al., 2017; Sermet et al., 2019) have provided the means to assess these anatomical predictions on a functional level, albeit largely *in vitro* in brain slices, and it has become clear that HO TC inputs provide input both excitatory and inhibitory neurons distinct from that provided by FO TC inputs.

### Cell-Type-Specific Innervation

#### Excitatory Neurons

HO TC inputs provide direct excitation to both L2/3 and L5 pyramidal neurons (Figure 1 green), although different degrees of cell-type-specific excitation are seen across cortical regions. In S1, HO POm fibers evoke EPSPs in excitatory neurons across all cortical layers (Audette et al., 2017; Sermet et al., 2019), with the largest responses in L5A pyramidal neurons (Bureau et al., 2006; Audette et al., 2017; Sermet et al., 2019), which is sufficient to evoke robust spiking (Audette et al., 2017). Audette et al. (2017) also report significant but smaller input to L2/3 pyramidal neurons *in vitro*. While earlier reports found little direct HO input to L5tt neurons (Petreanu et al., 2009), small but significant inputs were reported by Audette et al. (2017); Sermet et al. (2019). A main point is that POm only provides weak inputs to L4, the main cortical recipient layer of FO TC inputs (Audette et al., 2017; Figure 1 blue). Similarly, in the auditory system, projections from HO MGd to A1 L1 equally excite pyramidal neurons in L2/3 and L5 (Pardi et al., 2020). In HO nuclei targeted by PFC, the situation is different (Collins et al., 2018): excitatory inputs to L2/3 pyramidal neurons by HO MD are more than ∼3x greater than those to L5A neurons, while inputs from HO VM to both layers are comparably strong, suggesting that MD preferentially activates superficial neurons in L2/3. The relevance of L3 MD-PFC is further evidenced by the finding that the MD also drives disynaptic inhibition in L3 of medial PFC through excitation of PV interneurons, tightening the time window during which PFC pyramidal neurons can fire (Delevich et al., 2015).

Optogenetic manipulations of HO TC inputs *in vivo* have provided some evidence for how these inputs impact different cortical cell types in the intact brain. Gambino et al. (2014) find that HO POm evokes long-lasting NMDA-dependent plateaus in L2/3 pyramidal neurons, while (Mease et al., 2016a) show that HO POm projections provide long-lasting depolarizations in L5 neurons and enhance sensory responses *in vivo*, and this effect is even stronger under awake conditions (Zhang and Bruno, 2019). The recently proposed embedded ensemble encoding (Antic et al., 2018) theory suggests that ensembles of neurons experiencing a synchronized somatic depolarization are in a transient “prepared state” to respond with precise spike timing to additional inputs. Given this evidence for HO TC induction of sustained depolarizations, HO thalamus could play a role in coordinating such transient ensembles of “prepared” neurons and sensitizing the cortex to additional synaptic inputs. One experimental difficulty in assessing HO TC’s impact *in vivo* that mass optogenetic excitation and inhibition does not lend itself to physiological stimulation patterns and it is likely that more naturalistic interventions will reveal nuances of the effect of HO TC projections–for example, the use of step-opsins by Mukherjee et al. (2020) to show that enhancement of MD thalamus led to inhibition dominating activity in PFC.

#### Interneurons

HO-thalamus innervation of specific interneuron types in cortex (Figure 1) appears to be key to understanding the functional impact of HO TC inputs, with several recent studies taking advantage of molecular markers for different interneuron populations. In particular, in S1, POm HO TC inputs provide strong excitation to PV interneurons in L5a and L2/3 but little direct input to SOM interneurons (Audette et al., 2017; Sermet et al., 2019; Williams and Holtmaat, 2019). Thus HO TC provides disynaptic inhibition via PV to L5a pyramidal neurons, as well as direct excitation (Audette et al., 2017). A future direction will be to understand how PV interneurons encode naturalistic HO inputs *in vivo*, as PV neurons are particularly excitable, with low membrane time constants and high repetitive firing ability. Intriguingly Cruikshank et al. (2007) showed that, due to these single-cell properties, fast-spiking (presumed PV) interneurons are intensively driven by FO TC inputs. More recently, Jouhanneau et al. (2018) found that precise PV spiking can be evoked by unitary cortico-cortical EPSPs. Although HO TC to PV neuron encoding remains to be assessed, the strong synaptic drive in combination with high post-synaptic temporal precision suggests PV neurons may be able to follow high-frequency information in HO spike trains.

Interneurons in L1 are increasingly appreciated as targets of HO TC inputs, although direct comparison across studies is somewhat challenging due to variations in exact methodology and specificity of classification. A small group of studies has begun to clarify the importance of differential HO TC innervation of 5HT3 neurons in layer 1, which include VIP and NDNF populations (Schuman et al., 2021), particularly suggesting roles in disinhibition of deeper layers. In S1, 5HT3 interneurons receive direct HO POm input which evokes spiking *in vitro* (Audette et al., 2017). Notably, while somatostatin-positive (SOM) interneurons receive little direct HO TC input (Audette et al., 2017; Sermet et al., 2019; Williams and Holtmaat, 2019) find that PO excitation of VIP interneurons disynaptically inhibits SOM neurons. Similarly, Anastasiades et al. (2021) show that in PFC, HO MD projections directly target VIP neurons in L1, which then inhibit SOM neurons. These studies point to HO TC disinhibition of the pyramidal targets of SOM neurons, with possible implications for network synchronization, i.e., via Martinotti neurons’ interactions with L5tt discussed in the previous section. Finally, results from two different cortical regions demonstrate a role for neurogliaform/NDNF interneurons’ interaction with HO TC inputs in controlling pyramidal neuron excitability. Pardi et al. (2020) find that neurogliaform interneurons provide presynaptic inhibition of the HO MGd terminals involved in learning and triggering long-lasting NMDA potentials. Anastasiades et al. (2021) show that L1 NDNF interneurons in PFC are innervated by HO VM and inhibit SOM interneurons, but also act to block VM’s direct excitation of L5tt neurons’ apical tufts.

### HO Thalamus in Sensory Processing and Cognition

Cell-type-specific circuit interventions have revealed roles for HO thalamus in conveying signals important for learning, perception, and behavioral salience. Recent *in vivo* studies in behaving animals are also providing insight into the qualitative content of these HO thalamus signals, supporting roles for HO TC in promoting awake cortical behavior patterns associated with learning, cognitive flexibility, perception, and even consciousness.

In S1, studies by Holtmaat and colleagues provide a strong line of evidence for HO POm in generating long-term potentiation of intracortical synapses onto L2/3 pyramidal neurons via NMDA-dependent plateau potentials which depend on VIP-mediated disinhibition (Gambino et al., 2014; Williams and Holtmaat, 2019); these plateau potentials also provide a mechanism for cortical map plasticity (Pagès et al., 2021). In a multi-whisker sensory association task, Audette et al. (2019) found that training sequentially induces plasticity at HO POm TC synapses onto L5 and L2 pyramidal neurons, thereby increasing POm-driven spiking without changes in cortical single-cell properties. In an auditory associative learning task, Pardi et al. (2020) also demonstrated learning-related HO TC plasticity, finding that HO TC input to A1 transmits memory-related information which reflects task-specific relevance of sensory stimuli. El-Boustani et al. (2020) find that HO thalamus inputs show goal-directed modulation in mice trained in a whisker discrimination task; similarly (LaTerra et al., 2020) find that HO POm axons in S1 signal correct performance during goal-directed behavior and that inhibition of POm impedes task performance.

In the context of cognition, it is suggested that a generic role for the thalamus may be to coordinate and maintain cortical representations relevant for particular cognitive tasks (Halassa and Kastner, 2017; Nakajima and Halassa, 2017). For example, in an auditory-visual cue-switching attentional task, Schmitt et al. (2017) find that HO MD maintains PFC ensemble representations of task rules by control of functional connectivity. In the same behavioral paradigm, Rikhye et al. (2018) found that interactions between the PFC and HO MD provide a mechanism for cognitive flexibility to switch cortical representations, with MD thalamus encoding behavioral context. Although the specific contributions of L5 inputs to such cognitive tasks remain to be assessed, recent evidence suggests that L5-CTC loops may be key to conscious perception. For example, Suzuki and Larkum (2020) show that HO POm TC input enables L5tt dendro-somatic coupling necessary for awake activity patterns and robust somatic spiking, and that general anesthesia blocks this coupling. Although not specific to L5, Redinbaugh et al. (2020) find that in primates, activity in central lateral (CL) HO thalamus and deep-layer cortical neurons correlate with consciousness level; indeed, gamma stimulation in CL could rouse monkeys from anesthesia. These studies point to diverse but unexplored functions for HO nuclei in higher-level cognitive computations in the cortex.

We emphasize here that a core component of disentangling HO thalamus’s role in critical cortical function is quantification of how patterns of L5tt spiking are selected and transmitted back to the cortex. Finally, understanding the impact of specific patterns of HO neuron activity in cortex will require combining emerging knowledge of cell-type-specific TC synaptic dynamics with the state-dependent, nonlinear dendritic integration properties of cortical neurons, along with detailed microcircuit connectivity patterns.

## The Clinical Relevance of L5tt-HO CTC Networks

### The Pathological Role of L5-Originating CTC Networks in Pain

While further work is required to assess the underlying mechanisms and functionality of L5-originating CTC networks, recent studies have emphasized their potential clinical relevance (see Table 2). Among these, an increasing number of studies highlight the importance of these CTC networks in processing nociceptive information in both acute and chronic pain states. For example, spared nerve injury (SNI) in rodents- a model of neuropathic pain–results in a three-fold increase in Ca^2+^ activity in the somata of L5 pyramidal neurons in S1, as well as an increase in dendritic spine Ca^2+^ activity (Cichon et al., 2017). However, this study did not assess the impact of enhanced cortical activity on HO thalamic nuclei. Indeed, the development of chronic pain is associated with several maladaptive alterations in S1 and other cortical regions (e.g., hyperexcitability, somatotopic reorganization), but there remains a need to characterize how these alterations specifically influence CTC networks, especially given that these cortical alterations typically correlate with the degree of mechanical allodynia (i.e., a painful sensation resulting from typically innocuous mechanical stimuli) (Tan and Kuner, 2021). The relevance of these cortical alterations, and their potential impact on HO nuclei and CTC networks, is further exemplified by the fact that targeting S1 alterations beneficially alters pain trajectories (Flor et al., 2001; Moseley and Flor, 2012; Cichon et al., 2017). Hyperactive states in L5 neurons have also been observed in other cortical areas under inflammatory states–for instance, a study employing a peripheral inflammatory mouse model found transient hyperactivity in L5 pyramidal neurons in the frontal/motor cortex (Odoj et al., 2021). However, this study did not observe L5 hyperactivity in S1, so it is unclear whether the frontal/motor cortex alterations are pain-relevant.

Cichon et al. (2017) further investigated the role of inhibitory circuits in the development of S1 alterations, specifically, the source of L5 hyperactivity in chronic pain. It was shown that 1 month following SNI induction, somatostatin (SOM) interneuron activity, which regulates somatic and dendritic pyramidal cell activity, was reduced by half. Likewise, PV interneuron activity was reduced. In part, these findings were the result of a 90% increase in vasoactive intestinal peptide (VIP)-expressing interneuron activity in SNI animals, which directly inhibit SOM and PV interneurons. The SOM contribution to L5 pyramidal cell hyperactivity was confirmed through selective SOM cell activation, which decreased L5 dendritic and somatic Ca^2+^ activity and prevented the development of mechanical allodynia (Cichon et al., 2017). However, activation of PV interneurons did not alter mechanical thresholds for pain, perhaps because these cells predominantly synapse perisomatically, or because they provide only brief inhibition of somata (Cichon et al., 2017). Given the specific interaction of HO TC inputs with PV and VIP interneurons covered in the previous section and the plasticity of these connections (Audette et al., 2017, 2019; Williams and Holtmaat, 2019), it may be productive to further assess how HO TC inputs contribute to maladaptive cortical plasticity related to chronic pain.

It may be that L5 alterations in part contribute to changes observed in HO nuclei during chronic pain. For example, the PO nucleus, which is involved in pain processing, displays higher spontaneous firing rates and greater responses to both noxious and innocuous peripheral stimuli in a chronic pain state (Perl and Whitlock, 1961; Whitlock and Perl, 1961; Casey, 1966; Mao et al., 1993; Masri et al., 2009; Park et al., 2018). Another relevant HO nucleus is the MD, which mediates affective aspects of pain and is similarly hyperactive in chronic pain states. However, as with the PO, it is not understood if these changes in part stem from CTC alterations at the level of the cortex (Rinaldi et al., 1991; Wang et al., 2007; Whitt et al., 2013; Mitchell, 2015). Reinforcing its role in affective components of pain, optogenetic activation of MD inputs in the anterior cingulate cortex (ACC) elicits a conditioned place-aversion in the SNI model and chemotherapy-induced neuropathy, but intriguingly, direct inhibition of L5tt ACC projection neurons to the MD nucleus also produces this effect (Meda et al., 2019). In addition, inactivation and lesioning of the MD nucleus both resulted in a reduction in thermal and mechanical hyperalgesia in a rodent model of neuropathic pain (Saadé et al., 2007). Overall, while further effort is required to assess the overall role of CTC networks in pain, evidence suggests that both HO-projecting L5tt cells and HO thalamic nuclei are altered in pain states, and that targeting these alterations may serve to benefit pain trajectories.

### The Pathological Role of CTC Networks in Auditory Disorders

In the auditory system, comparable to the findings in chronic pain, Asokan et al. (2018) observed a hyperactive state of L5 projection cells in the auditory cortex following noise-induced damage to cochlear afferents, and this effect was sustained for several weeks (Asokan et al., 2018). Specifically, this L5 potentiation–which represents a form of compensatory plasticity–was observed in projections to the IC, but the same study also found axon collaterals to the HO MGBd nucleus (Asokan et al., 2018). Alterations in inhibitory networks are also implicated in this L5 pathology–for instance, PV interneuron-mediated intracortical inhibition is significantly reduced for at least 45 days following cochlear synaptopathy (Resnik and Polley, 2017). Increased sensory gain is a characteristic finding across noise-induced hearing-loss pathologies (e.g., tinnitus), and it is proposed that L5 projection neurons are responsible for driving hyperexcitability and strong coupling across tinnitus-associated brain networks (Asokan et al., 2018). As with the pain example discussed above, the impact of such pathological L5 activity on auditory HO nuclei remains to be assessed.

### The Pathological Role of CTC Networks in Cognitive/Behavioral Dysfunction

In addition to the apparent involvement of CTC networks in sensory modalities, recent work has alluded to their relevance to pathologies characterized by cognitive and behavioral dysfunction, such as schizophrenia (SZ) and Alzheimer’s disease (AD). Studies have demonstrated the involvement of the MD in these dysfunctions, and there is human, rodent and primate evidence that they can be elicited by damaging this thalamic nucleus (Mitchell et al., 2007, 2014; Mitchell and Gaffan, 2008; Mitchell, 2015; Perry et al., 2021). For instance, monkeys with damage in the MD display impairments in complex associated learning tasks (Mitchell et al., 2007). MD damage-associated impairment has been postulated to result from disruption of the influence of the MD nucleus on the PFC, since the MD nucleus and PFC are reciprocally connected within specific subdivisions (e.g., the pars parvicellularis of the MD is reciprocally connected to the dorsolateral PFC), but this view is contested (Mitchell, 2015; Collins et al., 2018). Specifically, the MD has been shown to activate cortico-cortical projections in layers 2 and 3 in the PFC (Collins et al., 2018). Further work on thalamic innervation of associative brain structures has shown that enhanced excitability in the MD elicits suppression of PFC excitatory neurons (Mukherjee et al., 2020).

Moreover, it was found that hypoxic-like damage to the PFC results in enhanced theta-frequency coherence between the MD and the PFC, as well as an increase in the frequency of bursting in MD neurons, while subsequent knockdown of T-type calcium channels (Cav3.1) in the MD nucleus decreased theta-frequency coherence and attenuated associated symptoms (e.g., frontal lobe seizures and locomotor hyperactivity). The authors propose that the observed neurological and behavioral abnormalities result from impaired thalamocortical feedback between the PFC and the MD, driven by the activation of thalamic T-type calcium channels (Kim et al., 2011). Moreover, abnormalities have been reported in HO nuclei in SZ patients, including reductions in the volume and activity of the MD nucleus and the PuV. This may disrupt transthalamic networks and account for schizophrenia-associated cognitive impairments (Sherman, 2017). In fact, Parnaudeau et al. (2013) show that pharmacogenetic inhibition of the MD nucleus disrupts MD-PFC synchrony in the beta range, causing cognitive impairment with relevance to SZ. Similarly, there is evidence that CT dysfunction contributes to cognitive and behavioral impairments observed in AD (Jagirdar and Chin, 2019).

In addition to these findings, it is increasingly understood that dendritic integration in pyramidal neurons, which plays essential roles in sensory processing and cognition, is disrupted in a range of neurodevelopmental disorders (Nelson and Bender, 2021). For example, autism spectrum disorder is associated with genetic changes that elicit functional, morphological, and organizational alterations in L5, but it is not understood if and how these cortical changes affect the rest of the CTC network (Nelson and Bender, 2021).

## Discussion

The thalamocortical field has clearly moved on from the historical view of the thalamus as simply a passive provider of input to the cortex, although this view is still surprisingly entrenched in general neuroscience literature. As comprehensively framed by Sherman and Guillery (2006); Sherman (2016), any adequate theory of cortical function must include active, dynamic, and iterative information processing by the thalamus. With unique L5tt input and output connectivity patterns in combination with distinct synaptic and intrinsic properties, HO thalamic nuclei are well-suited to support top-down information transformation and exchange critical for novelty detection and prediction (Keller and Mrsic-Flogel, 2018) or propagation of learning-related signals (Chéreau et al., 2021).

Here, we have sought to present a broad overview of L5tt corticothalamic information transfer to HO thalamus and back to the cortex. Rather than attempting comprehensive coverage, we have highlighted mechanistic properties and functional findings underscoring the point that insights from FO nuclei may not transfer well to HO nuclei as points of orientation for neuroscientists new to the vast, complex thalamocortical literature. Although we have attempted to balance findings across sensory modalities and HO regions where data are present, many recent illuminating studies were done only in single regions–for example, many of the very recent cell-type-specific functional insights about HO TC inputs are from the somatosensory system. Lastly, in focusing largely on studies using cell-type-specific and/or experimental methods tractable in rodents, by necessity most of the presented information comes from experiments in mouse and rat models.

A key point is that in comparison to the relatively rich theoretical framework focused on cortical function, further subcortical processing of L5tt signals has been comparatively neglected. Cortico-centric models have yet to be fully integrated with the transthalamic communication model (Sherman and Guillery, 2011)–we emphasize that a central missing piece is how exactly L5tt signals are processed and encoded in HO thalamus. Whatever the modality-specific information L5tt spike trains send to HO thalamus, these signals will be further transformed by a heady blend of strong synaptic depression, the nonlinear input/output properties of single HO thalamic neurons tuned by dynamic L6A and TRN-driven excitation and inhibition, and finally, integration with other subcortical drivers and strong extrathalamic sources of inhibition.

Cast in this light, it is not particularly surprising that the question of what HO thalamus encodes is currently unanswered–but solving this puzzle appears increasingly central to understanding cortical network function underpinning cognition and perception. We have emphasized the viewpoint that it may be more productive to consider HO thalamus’s encoding of driving cortical signals from L5tt, rather than any particular parameterization of raw sensory stimuli. Given the evidence for HO integration of L5tt inputs with non-cortical drivers and various neuromodulatory signals, this viewpoint is clearly an oversimplification–but possibly still a useful beginning step in linking L5tt drive of HO thalamus to general theories of cortical computation. More specifically, we hypothesize that HO thalamus may be able to simultaneously detect and transmit distinct patterns of L5tt synchrony and high-frequency spiking, and that these signals may have cell-type-specific functions in the cortex depending on HO TC postsynaptic targets. In the future, these ideas could be tested by combining high-yield electrophysiology approaches, cell-type-specific interventions, and recent advances quantifying selective information transfer between brain regions, such as the “communication subspace” scheme described by Semedo et al. (2019).

Although the synaptic and intrinsic mechanistic pieces appear to exist for an adaptive information-dense corticothalamic code from L5 to HO thalamus, there are still significant experimental and theoretical efforts to be made. Computational modeling would expedite understanding of how HO thalamus encodes cortical L5 spike trains, but based on our survey of ModelDB (Hines et al., 2004), very few models of HO thalamic neurons have been published (Golomb et al., 2006; Desai and Varela, 2021) and certainly none with the level of biophysical detail as recent modeling efforts focused on FO neurons (e.g., Connelly et al., 2016; Iavarone et al., 2019). Such efforts could boost further assessment of the relationship between bursting in L5 and HO thalamus, as it is clear that bursts can play privileged roles in both transmitting information and engaging plasticity mechanisms (Larkum, 2013; Crunelli et al., 2018; Zeldenrust et al., 2018b; Payeur et al., 2021). In the future, it will be important to expand existing network models of thalamocortical interactions which are mainly but not always (see Golomb et al., 2006) based on FO data to include HO and FO distinctions in driving input, intrathalamic inhibition, and intrinsic properties which we have attempted to summarize here.

Finally, studies have begun to shed light on the clinical relevance of HO-thalamus CTC pathways, as a range of disruptions along these pathways, especially in HO nuclei, have been implicated in pathologies including chronic pain, SZ, and AD. However, present studies have largely concentrated efforts on characterizing either cortical or HO thalamic dysfunction in pathological contexts, but do not consider the interregional relationships. As such, further efforts are merited to transfer recent fundamental insights from sensory processing and cognition to pathology in L5tt-HO thalamus circuits, in particular studies that assess CTC pathways in their entirety. In the future, improving our understanding of these pathways in both pathological and non-pathological settings may serve to facilitate the identification of novel therapeutic targets and inform clinical strategies.

## Author Contributions

RM: conception, planning, and funding acquisition. RM and AG: research, writing, and editing. Both authors contributed to the article and approved the submitted version.

## Conflict of Interest

The authors declare that the research was conducted in the absence of any commercial or financial relationships that could be construed as a potential conflict of interest.

## Publisher’s Note

All claims expressed in this article are solely those of the authors and do not necessarily represent those of their affiliated organizations, or those of the publisher, the editors and the reviewers. Any product that may be evaluated in this article, or claim that may be made by its manufacturer, is not guaranteed or endorsed by the publisher.

## Abbreviations

AHP: afterhyperpolarization
AD: Alzheimer’s disease
ACC: anterior cingulate cortex
APT: anterior pretectal nucleus
CT: corticothalamic
CTC: cortico-thalamo-cortical
MGBd: dorsal aspect of medial geniculate body
EPSP: excitatory postsynaptic potential
EPSC: excitatory postsynaptic current
FO: first-order
HO: higher-order
I_*H*_: hyperpolarization-activated cation current
LP: lateral posterior nucleus
L5: layer-V
L6: layer-VI
MD: mediodorsal nucleus
PV: parvalbumin
POm: posterior medial nucleus
PO: posterior nucleus
PPC: posterior parietal cortex
PFC: prefrontal cortex
A1: primary auditory cortex
M1: primary motor cortex
S1: primary somatosensory cortex
V1: primary visual cortex
PuV: pulvinar nucleus
A2: secondary auditory cortex
S2: secondary somatosensory cortex
SOM: somatostatin
SNI: spared nerve injury
SZ: schizophrenia
TT: thick-tufted
I_*T*_: transient low-threshold calcium current
VIP: vasoactive intestinal peptide
ZI: zona incerta.
